# Bibliometric analysis of research trends and prospective directions of *Akkermansia muciniphila* from 2010 to 2024

**DOI:** 10.3389/fmicb.2025.1569241

**Published:** 2025-04-16

**Authors:** Yanan Wang, Jiahui He, Simin Chen, Xinyi Lv, Jiayi Chen, Kaiyue Ru, Xiao Liang, Meng Mao, Yuehan Song

**Affiliations:** ^1^Beijing University of Chinese Medicine, Beijing, China; ^2^Qianfoshan Hospital in Shandong Province, Jinan, China

**Keywords:** bibliometrics, visual analysis, *A. muciniphila*, gut flora, frontiers

## Abstract

**Background:**

*Akkermansia muciniphila* (*A. muciniphila*) is an emerging probiotic with potential impact on human health, and there is a growing interest in this area, but an overall analysis of research trends is lacking. This study conducted a detailed bibliometric analysis and visualization of *A. muciniphila* research to examine the current research status, hotspots, and trends, aiming to inform future research directions.

**Methods:**

This study utilized the Web of Science database to search research on *A. muciniphila* from 2010 to 2024. Bibliometric analysis was conducted using CiteSpace and VOSviewer software to generate yearly publication trends, contributions by countries, institutions, and distinguished researchers, as well as key themes and influential researches. This analysis aimed to visualize and explore the literature over the past 15 years, guiding future researches and identifying gaps in the field of intestinal flora in *A. muciniphila*.

**Results:**

We searched a total of 4,423 related publications. Wei Chen, Willem de Vos and Patrice D. Cani are the primary contributors to *A. muciniphila* ‘s research. The top contributing countries and institutions are China, the United States, South Korea, Spain, and Italy, with research centers such as the Chinese Academy of Sciences, Zhejiang University, the University of Copenhagen, and the University of Helsinki being the main contributors. Current research hotspots focus on the molecular biology of *A. muciniphila*, such as its role in intestinal barrier maintenance, immune response, and its potential for regulating and treating digestive and metabolic diseases, such as cancer, fatty liver disease, inflammatory bowel disease, etc., through bile acid metabolism, extracellular vesicles, and insulin resistance.

**Conclusion:**

Our study synthesizes current research on *A. muciniphila* in various disease areas and suggests enhancing collaboration among countries, institutions, and authors to advance *A. muciniphila*—related clinical and basic research, explore its efficacy in a variety of diseases and the effects of commonly used clinical medications on *A. muciniphila*, to fill the research gaps in the current field, and to provide valid evidence for the development of *A. muciniphila* as a novel probiotic supplement.

## 1 Introduction

The gut hosts hundreds of millions of flora, and in recent years, scientific research has highlighted the crucial symbiotic relationship between gut microbiota and the host, demonstrating its significant roles in regulating nutrition, metabolism, and immunity (Maynard et al., 2012; Stepanova and Aherne, 2024). *A. muciniphila*, a Gram-negative anaerobic bacterium from the intestinal flora’s warty microflora phylum, is a promising probiotic in next generation probiotics (NGPs) (O’Toole et al., 2017). It engages in cellular communication via metabolites like short-chain fatty acids and proteins on extracellular vesicles and outer membranes, such as *Akkermansia muciniphila* outer membrane protein 1100 (Amuc_1100), due to its proximity to the intestinal wall (Gu et al., 2021). *A. muciniphila* degrades mucin, enhancing the intestinal mucus layer’s thickness, thereby protecting the mucus barrier. It also induces intestinal stem cell differentiation into secretory cells, boosting insulin sensitivity and reducing inflammation, garnering significant attention in recent years (Cani et al., 2022; Derrien et al., 2011; Xu et al., 2023). Although *A. muciniphila* only account for 3–5% of the intestinal flora (Belzer and de Vos, 2012), a large number of studies have demonstrated that *A. muciniphila* has demonstrated excellent therapeutic potential in the treatment of inflammatory bowel disease, obesity (Schneeberger et al., 2015), atherosclerosis (Li et al., 2016), diabetes (Shin et al., 2014), cancers (Cani et al., 2022), chronic liver disease (Keshavarz Azizi Raftar et al., 2021), progeria (Blacher et al., 2019), premature aging (Bárcena et al., 2019) and other diseases by improving the intestinal barrier function in the host, modulating the immune system response, participating in the intestinal and hepatic axis signal transmission, and promoting the level of metabolism (Wade et al., 2023; Chen et al., 2021; Wang et al., 2020).

In recent years, *A. muciniphila* have made remarkable progress in research, with exponential growth in their association with gut microbiota homeostasis, metabolic diseases and immune regulation. However, there is still a lack of systematic research in this field, and traditional reviews are easily limited by the subjective knowledge of researchers, while fragmented meta-analysis is difficult to reveal the evolution of the knowledge structure in this field. Bibliometrics, as a core branch of scientometrics, is dedicated to evaluating the quantitative characteristics, trends and academic impact of scientific literature (Zhou et al., 2022; Liao et al., 2023), and can objectively quantify the evolution trajectory, knowledge base and research frontiers of scientific literature through mathematical statistics and visualization techniques (Chen, 2017). Compared with traditional reviews, this method has three unique advantages: (1) based on the search of massive literature data, it can identify the hidden associations that are difficult to be detected by human readers; (2) revealing the structural characteristics of the knowledge base of disciplines through the analysis of the co-citation network; (3) capturing the migration patterns of research hotspots. Nowadays, it has been successfully applied in many disciplinary fields. In this study, we innovatively constructed the first fifteen-year bibliometric mapping of *A. muciniphila* research. By integrating multidimensional methods such as country/institutional productivity analysis, author collaboration networks, literature co-citation timeline evolution, and keyword emergence detection, it systematically explains how the core academic community in the field is formed and evolved and the development trend, clarifies the current research priorities of *A. muciniphila* and predicts the future research directions (Li et al., 2022), and integrates the milestone studies that advance the discipline. The results of this study will provide shortcuts to field awareness for new researchers, assist senior scholars in locating opportunities for interdisciplinary collaborations, and provide data support for funding organizations to grasp the direction of strategic investments.

## 2 Materials and methods

### 2.1 Data sources and search strategies

**Figure 1:**
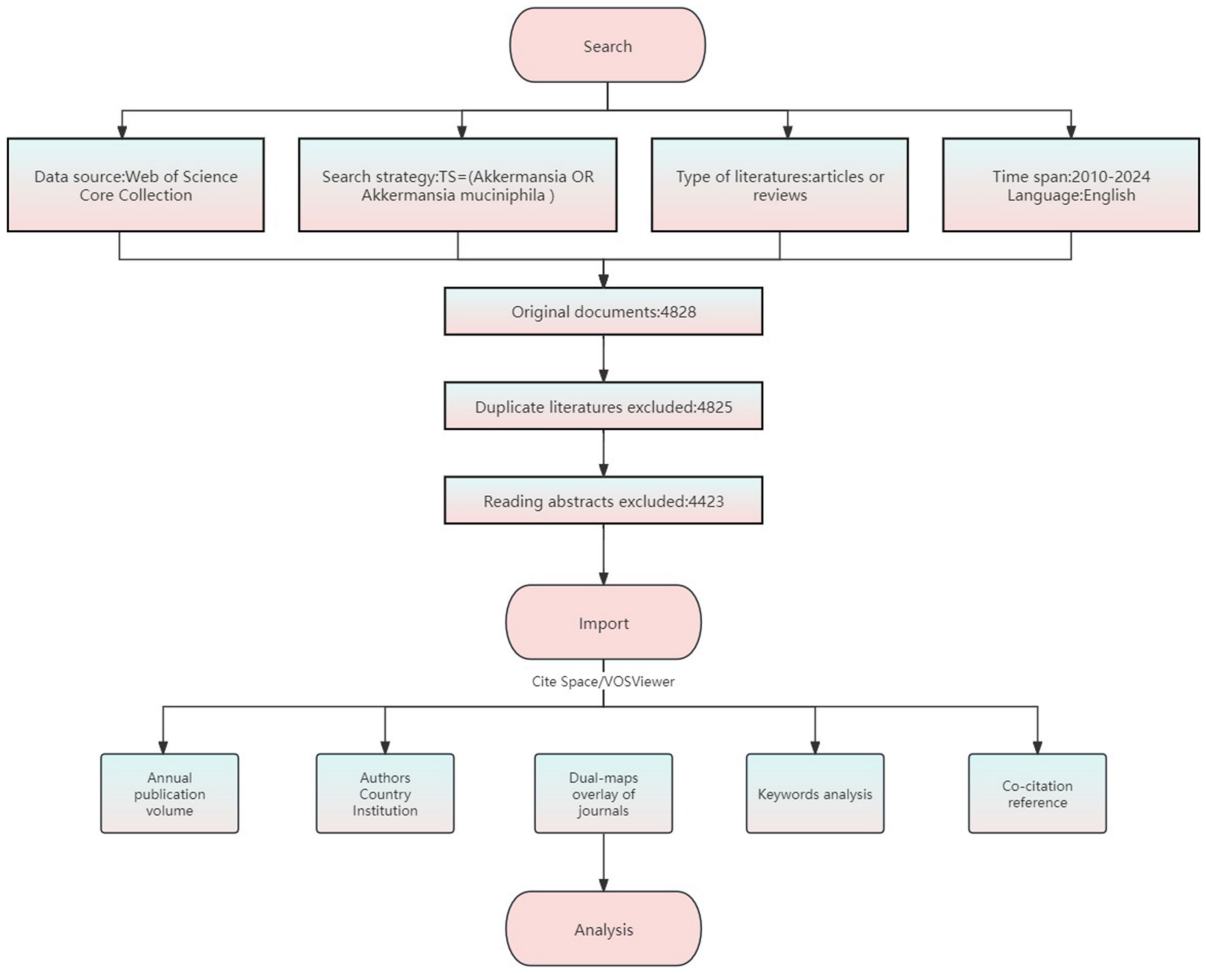
Flowchart for research.

Web of Science provides a standardized citation data format (RIS/Claim Text) that is compatible with mainstream bibliometric tools such as VOSviewer and CiteSpace; Covering a wider range of interdisciplinary journals such as microbiology, medicine, biotechnology, etc., it is considered the most complete and trustworthy bibliometric analysis database (Lyu et al., 2022). Therefore, this study was based on Web of Science Core Collection including SCIE (Science Citation Index Expanded), SSCI (Social Science Citation Index), A&HCI (Arts and Humanities Citation Index), ESCI (Emerging Sources Citation Index), Conference Proceedings Citation Index (CPCI), and Book Citation Index (BKCI) for data collection. The process is shown in Figure 1. Referring to the PubMed MeSH thesaurus, the core term “*Akkermansia muciniphila*” and “Akkermansia” were identified, and Boolean operators were used to formulate the search formula TS = (“*Akkermansia muciniphila*” OR “Akkermansia”), with the limitation language English, type Article or Review, retrieved studies from 2010 to 2024. To ensure data quality and format standardization, the “txt” file in Refworks format was imported into NoteExpress software for de-duplication and screening, and duplicated records were eliminated by using a literature management tool; and original studies that met the requirements for research topics related to the function, mechanism, or application of *A. muciniphila* were screened by reading. We further screened the articles or reviews that fulfilled the research topic related to the function, mechanism or application of *A. muciniphila* by reading them, and excluded letters, reviews, editorials, conference papers, books, corrections, news and retracted publications and duplicated publications. A final selection of 4,423 documents was made.

### 2.2 Visualization analysis tool and analysis methods

VOSviewer is a software tool developed by the University of Leiden suitable for processing large-scale data and constructing visual bibliometric maps (van Eck and Waltman, 2010; Chen, 2004); CiteSpace is a practical software for visual analysis of trends and patterns in the field that allows for the identification of new trends and developments in the field using dynamic network analysis techniques (Guzmán and Chen, 2016). This study utilized VOSviewer 1.6.20 software for institutional, journal, and keyword co-occurrence analysis, and CiteSpace 6.1.R6 software for author, country, and keyword clustering, emergence, and timeline analysis (Chen, 2006). The parameter settings are as follows: the selected time zone for data processing is from 2010 to 2024, set the time slice to 1, select k = 25 in the Selection Criteria section, adjust the topN to 50, and check the Pruning column with the cropping method of “Pathfinder” and “Purning sliced networks.” The term sources include “Title,” “Abstract,” “Author” and “Keywords,” etc. The node types include author, organization, keywords, etc. The rest of the settings are set as default. Among them, the calculation of citation burst intensity is based on Kleinberg’s burst detection algorithm, which identifies the abnormal growth pattern of citation frequency in the time series. This study implements it through CiteSpace, with parameters set as: minimum burst duration = 2 years, and significance threshold <0.05. During the analysis, synonyms were merged using the txt file find and replace function to improve the accuracy of the analysis, and three researchers verified and determined the consistency including the simultaneous existence of the same word with different cases, the co-existence of singular and plural forms of synonyms, and the co-existence of full names and abbreviated terms.

## 3 Results

### 3.1 Trends in annual volume of communications

**Figure 2:**
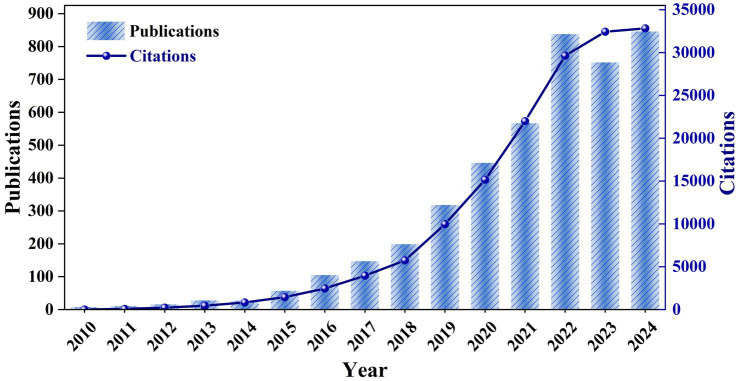
Trends in the number of publications and articles related to *A. muciniphila.*

The notable shifts in publication volume and citation frequency indicate research trends and heightened academic interest in the field. As shown in Figure 2, there were only a dozen or so relevant publications from2010 to 2014, indicating that the field was in its infancy, but the citation frequency rose from 456 to 837 in 2014, indicating that the existing studies began to be widely cited. Following extensive research on *A. muciniphila*’s role in metabolic health, obesity, diabetes, and the gut microbiome, the field experienced rapid growth from 2015 to 2024. During this period, annual publications increased from 57 to 846, and citations rose from 1,460 to 32,832, indicating ongoing advancements in this area of study. Even though the research focus has shifted under the influence of the COVID-19 pandemic, the exploration of the *A. muciniphila* field still continues to grow. Future research will likely focus on the mechanistic exploration of *A. muciniphila*, its clinical applications and its potential value in personalized medicine.

### 3.2 Author mapping analysis

**Figure 3:**
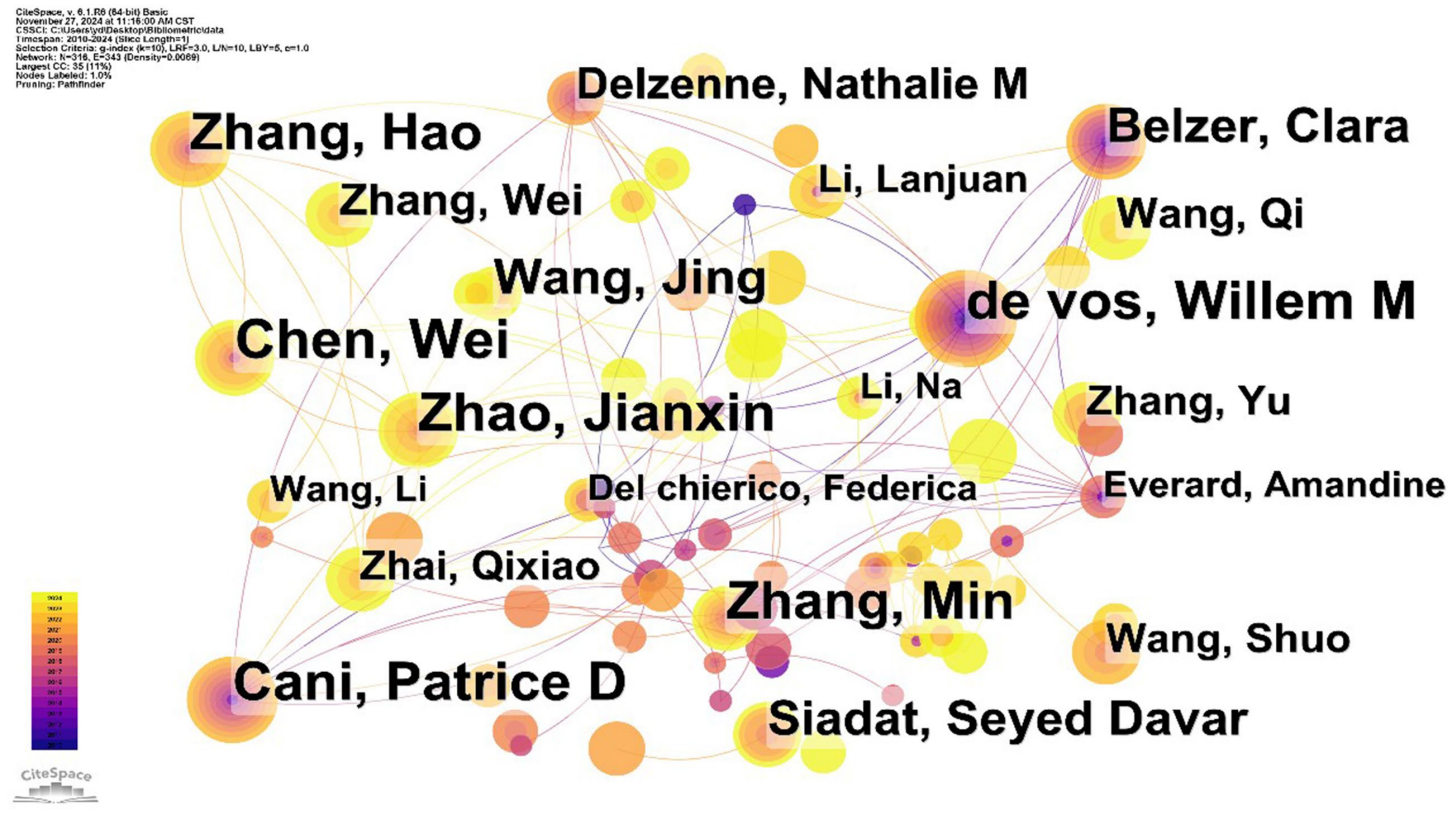
Co-occurrence of principal investigators of *A. muciniphila.*

Based on the analysis, Wei Chen leads in academic contributions on A. muciniphila with 41 publications, primarily exploring its connection to host metabolic health and its mechanisms in metabolic diseases like obesity and diabetes. Willem M. de Vos and Patrice D. Cani each made significant contributions with 36 publications. Willem de Vos, a prominent microbiome researcher at the Wageningen Microbiology Laboratory in the Netherlands, is recognized for isolating and naming the bacterium A. muciniphila from healthy adult feces (Derrien et al., 2004). In 2019, Patrice D. Cani’s team, based on previous animal studies, published the results of the first clinical trial of A. muciniphila, which demonstrated that supplementation with inactivated A. muciniphila significantly improved several metabolic indices in people with overweight or obesity and insulin resistance, triggering a discussion on the concept of “postbiotic” and its potential application (Depommier et al., 2019). In addition, Jianxin Zhao and Min Zhang, each with 36 and 35 publications, have made important contributions to the basic biological characterization of A. muciniphila. However, the mediator centrality of all the above authors was below 0.1, indicating that the research collaboration among them is still relatively fragmented (Figure 3).

### 3.3 Analysis of countries and institutions

**Figure 4:**
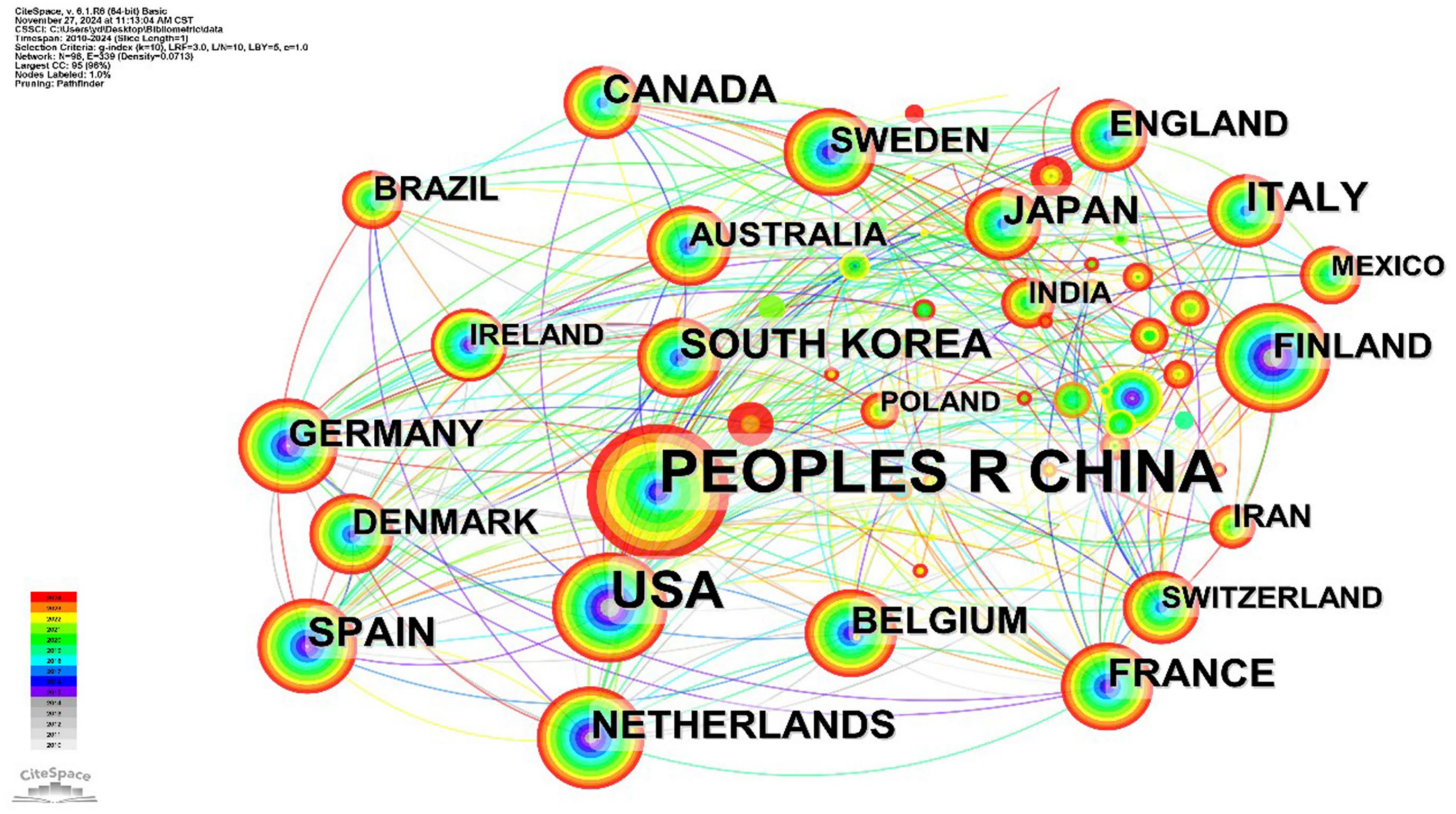
Country cooperation networks for *A. muciniphila.*

A network diagram illustrating collaboration among countries and institutions, represented as nodes, was created through visual analysis. As depicted in Figure 4, 98 countries/regions contributed to *A. muciniphila* research. China, with 2,292 publications, is at the top of the list and also plays a pivotal role in *A. muciniphila* research; the United States, as a country with sufficient research funding and facilities, follows with 753 publications, and most of the top institutions, led by Harvard University, Stanford University, and Johns Hopkins University, focus on *A. muciniphila* and metabolism, inflammation and the exploration of its mechanisms of action in intestinal immunity.

It is worth noting that the circles represent different countries, their size indicates the number of papers published in each country, the thickness of the links indicates the degree of cooperation between countries, and the different color of the aperture represents different publication times. In the top 10 countries for publications—China, United States, Korea, Spain, Italy, Canada, France, Japan, Netherlands, and Germany—the USA had the highest mediated centrality at 0.59, with China, France, and Germany following at 0.26, 0.24, and 0.21, respectively. Medical research on *A. muciniphila* has initially formed a transnational and interdisciplinary collaborative network. Despite varying publication outputs across countries, they have consistently advanced cutting-edge research in this field through cooperation and information sharing.

**Figure 5:**
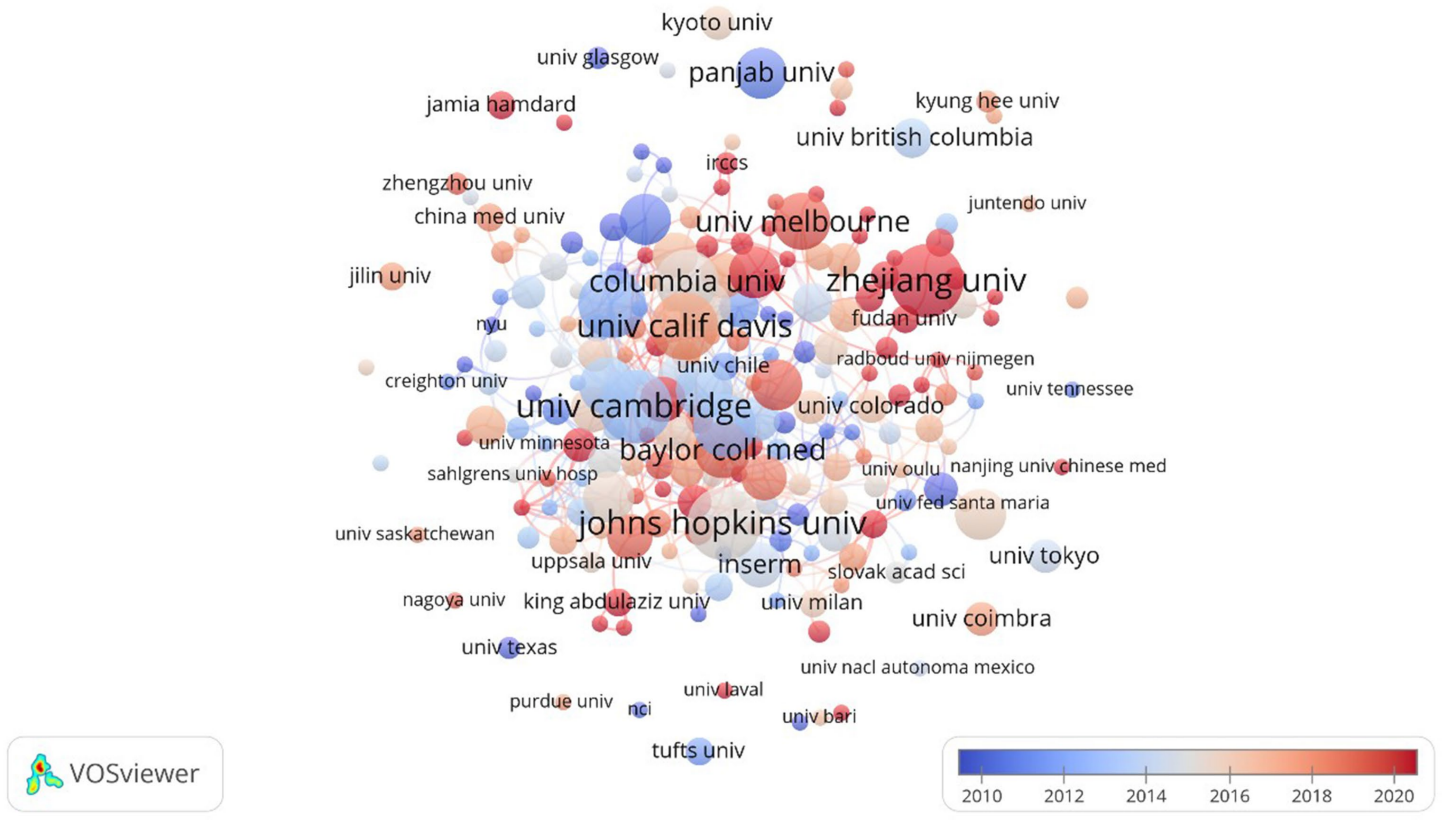
Map of medical research organizations of *A. muciniphila.*

As shown in Figure 5, Chinese research institutions, including the Chinese Academy of Sciences and Jiangnan University, both published 100 publications, followed by Zhejiang University with 97 publications. Shanghai Jiao Tong University has published 73 studies focusing on the clinical potential of *A. muciniphila*, particularly its role in metabolic and inflammatory bowel diseases. Southern Medical University published 57 articles investigating *A. muciniphila*’s role in the gut microbiome and its effects on the immune system. The University of Helsinki, Finland, with 60 articles, but the University of Wageningen focuses more on the promising applications of Akk in microbial agriculture. Despite having 50 publications, the University of Copenhagen exhibits the highest institutional centrality at 0.27. Other institutions with notable centrality include Zhejiang University (0.17), the Chinese Academy of Sciences (0.16), the University of Helsinki (0.15), Catholic University of Louvain (0.15), and Harvard Medical School (0.14).

### 3.4 Journal analysis

**Figure 6:**
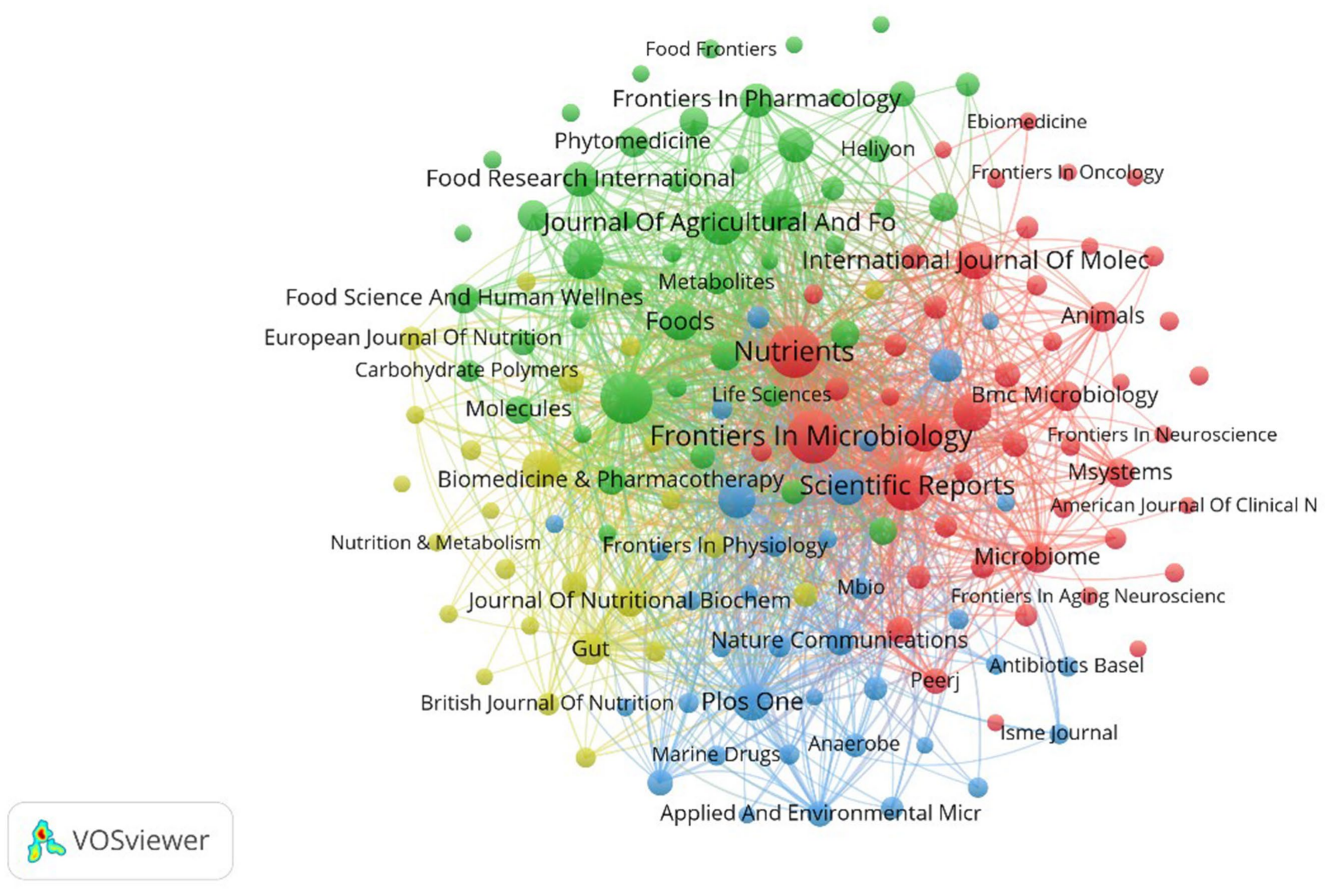
Map of medical research journal publications of *A. muciniphila.*

As shown in Figure 6, the font size of each journal corresponds to its number of publications, and the thickness of the lines indicates the level of collaborative effort between institutions in research. 157 academic journals are pivotal in disseminating *A. muciniphila* research within the medical field and advancing related studies. *Frontiers in Microbiology* dominates with 200 articles and 6103 citations, and the journal not only covers basic microbiology research, but also focuses on the role of microorganisms in health and disease. *Food & Function and Nutrients*, with 188 and 187 articles, respectively, focused on food science and nutrition, as *A. muciniphila* as a probiotic is closely related to diet, and *Scientific Reports*, an interdisciplinary open-access journal, ranked among the top with 135 articles and 6,647 citations. *The Journal of Agricultural and Food Chemistry* published 93 articles, *the Journal of Functional Foods* published 84 articles, and *Frontiers in Nutrition* published 83 articles, all highlighting the significance of dietary effects and microbiome interactions. *Frontiers in Cellular and Infection Microbiology* published 66 articles focusing on *A. muciniphila*’s impact on host immune responses and infection risk. Although the top international journals *Gut* (IF = 23.0) and *Nature Medicine* (IF = 58.7) published only 33 and 11 *A. muciniphila*-related papers, they reached the highest citation frequency of 11,391 and 5,964, which is the same as *Scientific Reports*, *Frontiers in Microbiology*. The influence of *A. muciniphila* is greater than that of *Scientific Reports*, *Frontiers in Microbiology*, etc.

### 3.5 Research hotspots analysis

#### 3.5.1 Keyword co-occurrence analysis

**Figure 7:**
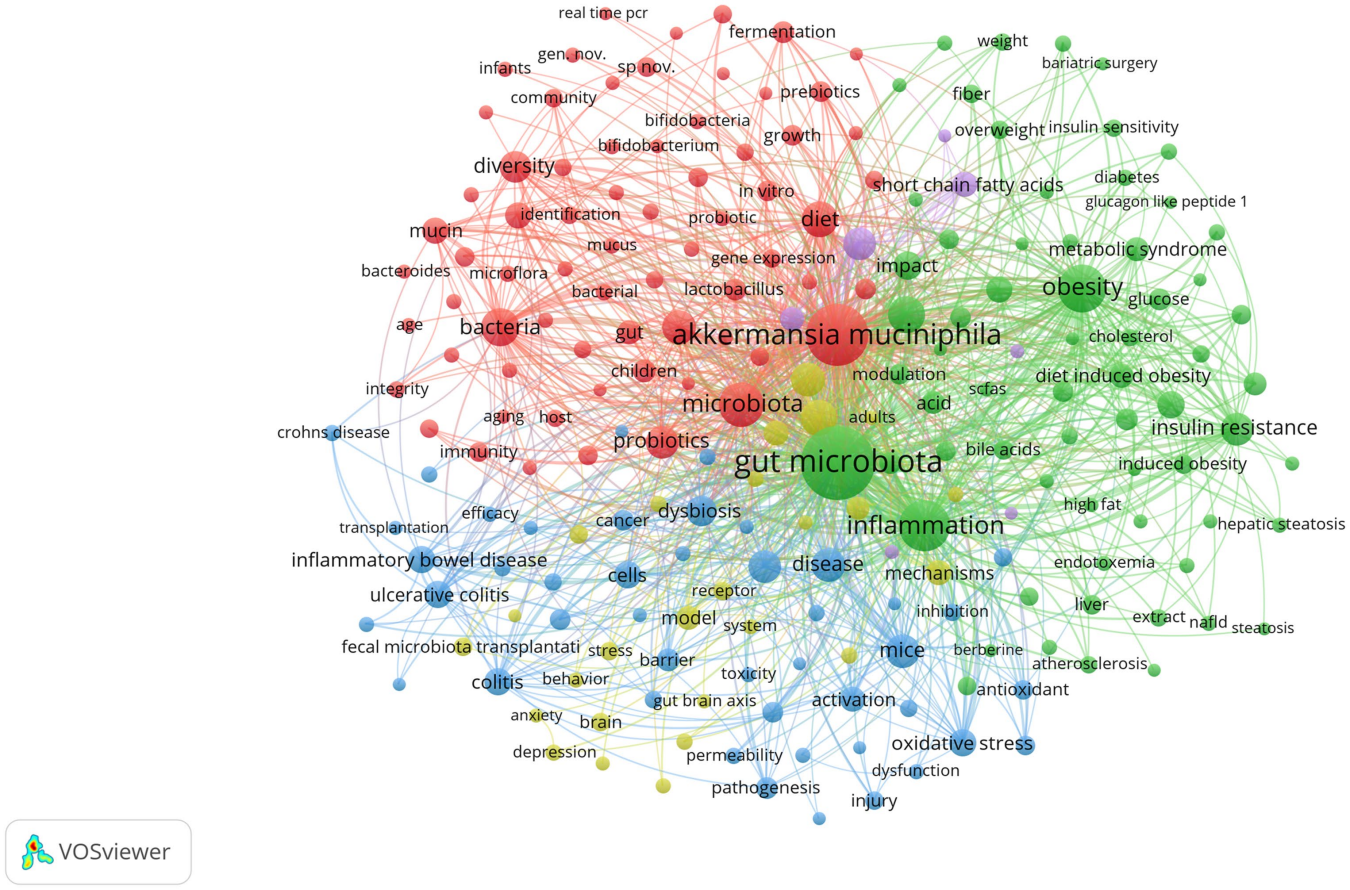
Keyword co-occurrence network for medical research on *A. muciniphila.*

The co-occurrence of high-frequency words reflects the research hotspots. The relational network of concurrent keywords is shown in Figure 7 illustrates the frequency of key terms in the research field. Each node represents a keyword, and the size of the node reflects the frequency of occurrence of the keyword in the literature; the larger the node, the higher the frequency of occurrence of the keyword. The colors of the nodes represent different topics or domains, and nodes with similar colors indicate that they have high relevance. The connecting line indicates the co-occurrence relationship between two keywords, the thicker the connecting line, the higher the frequency of co-occurrence of two keywords in the literature. Excluding the theme word “*Akkermansia muciniphila*” (frequency 1477), “Gut Microbiota” is the most common keyword (frequency 2677), whose diversity is essential for maintaining host health, and “Dysbiosis” (frequency 168) when it may lead to “Inflammation” (frequency 723) reaction, “Metabolic Syndrome” (frequency 138) and “Insulin Resistance” (frequency 294) states. The frequent mention of keywords like “Diet” (frequency 342) and “High Fat Diet” (frequency 178) highlights the significant role of diet in influencing *A. muciniphila* abundance and function. Specifically, diets high in dietary fiber notably enhance *A. muciniphila* levels, contributing to better metabolic health and weight management (Zhang et al., 2022). For example, “Diet-Induced Obesity” (frequency 136), whereas a high-fat diet not only leads to “Obesity” (frequency 495), while also modifying gut microbiome composition and function, thereby worsening metabolic issues. Other frequently mentioned keywords are *A. muciniphila* metabolites, specifically “Short Chain Fatty Acids” (frequency 191), and the related condition “Inflammatory Bowel Disease” (frequency 212).

#### 3.5.2 Keyword clustering analysis

**Figure 8:**
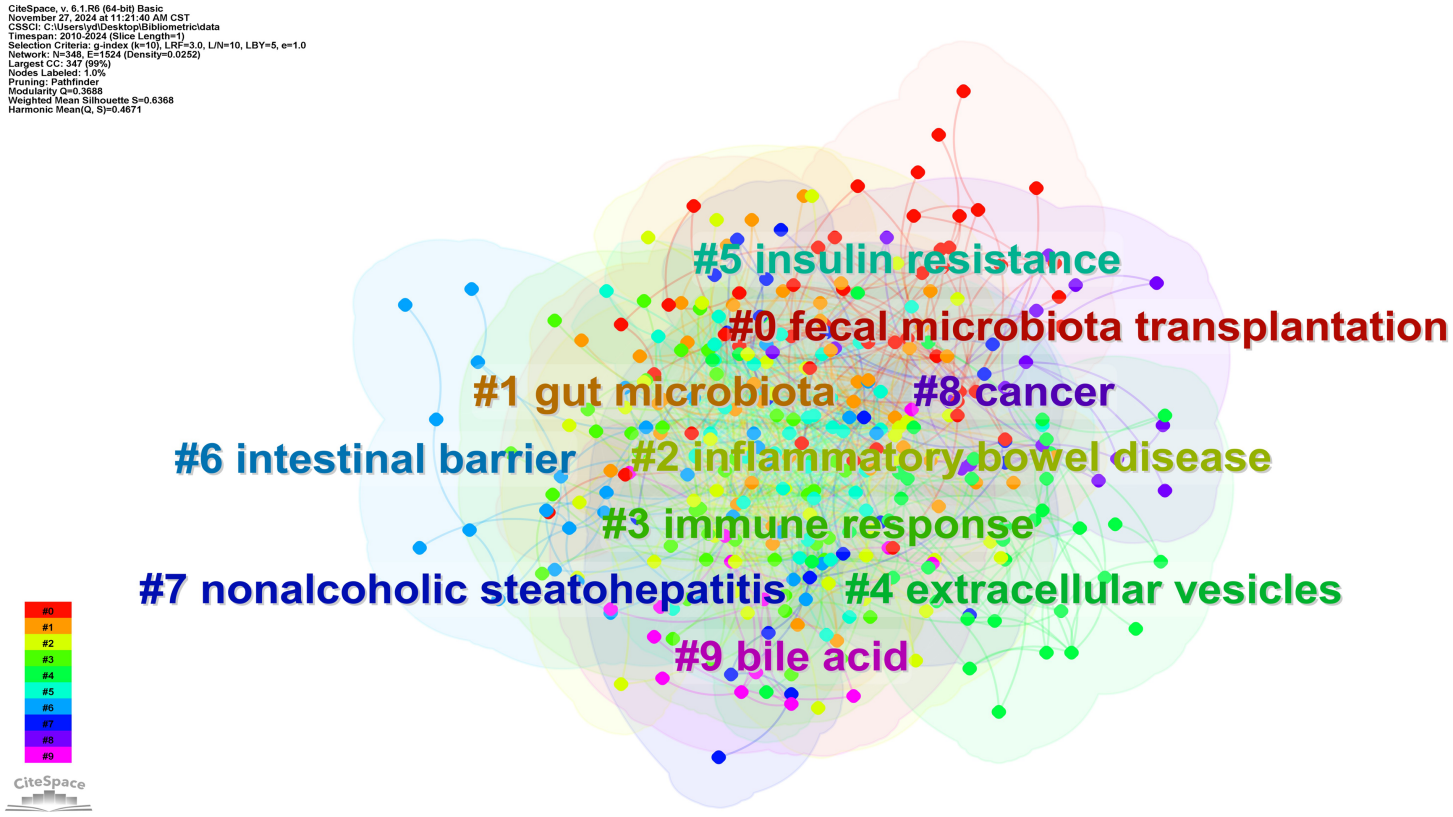
Clustering of medical research keywords of *A. muciniphila.*

Through CiteSpace analysis, we clustered the related literature by covering the number of keywords, silhouette index, year of clustering, and research content to reveal the trends and relevance of different research topics. Among the ten main clusters, fecal microbiota transplantation (#0) is a technique involving the transfer of fecal flora into a recipient’s gastrointestinal tract via enema or gavage. This method aims to modulate intestinal microecology and is a primary validated approach for external interventions to regulate the organism through intestinal flora (Vaughn et al., 2019). For example, the great potential shown in inflammatory bowel disease; inflammatory bowel disease (#2), nonalcoholic steatohepatitis (#7), and cancer (#8) constitute a category focusing on the therapeutic application of *A. muciniphila* in the field of disease; gut microbiota (#1), immune response (#3), extracellular vesicles (#4), insulin resistance (#5), intestinal barrier (#6), bile acid (#9) focusing on the study of mechanism of action on *A. muciniphila*. The above categorization is shown in Figure 8, where the studies are closely related and each color represents a cluster. Table 1 provides a detailed description.

**Table 1:** Keyword clustering analysis.

Cluster ID	Size	Silhouette	Label (LLR)
0	46	0.446	Fecal microbiota transplantation; gen. Nov.; infection; transplantation; meta analysis
1	45	0.715	Gut microbiota; intestinal microbiota; short-chain fatty acids; *Akkermansia muciniphila*; fecal microbiota
2	44	0.625	Inflammatory bowel disease; ulcerative colitis; gut microbiome; crohns disease; bacteria
3	43	0.572	Immune response; growth performance; 16 s rRNA; broiler; intestinal epithelial cells
4	41	0.628	Extracellular vesicles; colorectal cancer; diabetic nephropathy; brain; coliti
5	40	0.748	insulin resistance; metabolic syndrome; obesity; high fat diet; diet induced obesity
6	33	0.609	Intestinal barrier; intestinal flora; oxidative stress; extract; tea polyphenols
7	22	0.683	Nonalcoholic steatohepatitis; fatty liver; model; nonalcoholic fatty liver disease; animal models
8	18	0.755	Cancer; risk; high-throughput sequencing; inhibition; Alzheimer’s disease
9	15	0.787	Bile acid; gene; gut microbe; bile acids; gut microbiota

The Silhouette value is used as a quantitative indicator of cohesion and separation within a cluster (range −1 to 1). The closer the value is to 1, the higher the similarity of the keywords within the cluster and the more significant the separation from other clusters (Rousseeuw, 1987). Usually, a threshold value of >0.5 is considered to be structurally sound, and all the clusters in this study have a Silhouette value of >0.5, which indicates that the keywords within the clusters have a high degree of semantic consistency and the boundaries between the classes are clear, which is in line with the standard of high-quality clustering in bibliometrics.

#### 3.5.3 Analysis of keyword time trends

**Figure 9:**
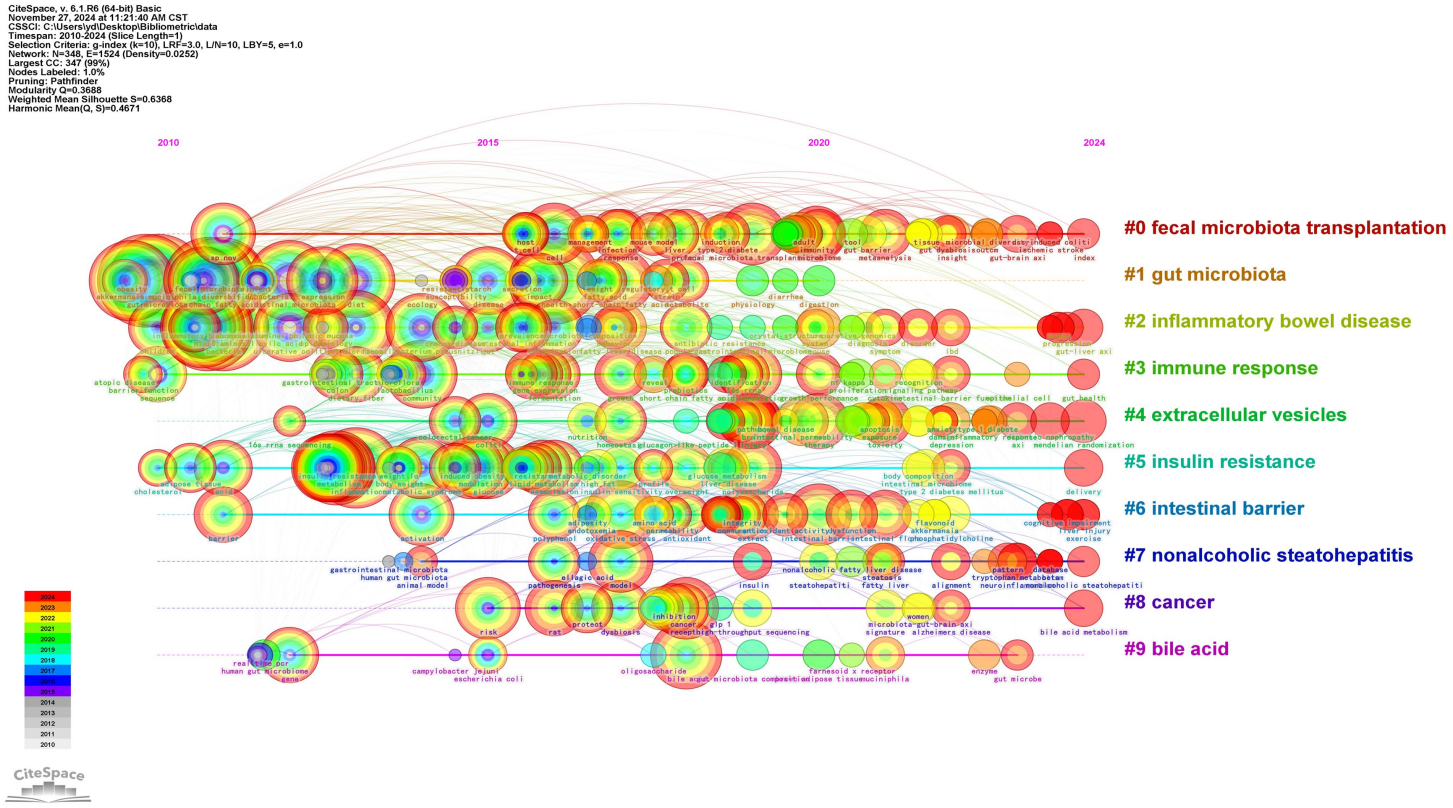
Timeline of medical research keywords for *A. muciniphila.*

The temporal trend analysis highlights the historical development and emerging hotspots in *A. muciniphila* research over the past 15 years. As shown in Figure 9, in CiteSpace, each node signifies an individual item, with its size denoting its importance or influence. Colored circles within the nodes represent citations of a specific article, where the overall size corresponds to the citation count and the color indicates the citation’s time period (Liu et al., 2019). Each horizontal line denotes a cluster, with smaller tag numbers signifying larger clusters. The node size reflects the frequency of co-citations, and the links indicate co-citation relationships. Node and line colors represent various citation years.

First, from 2010 to 2011, the keywords “gut microbiota,” “*Akkermansia muciniphila*,” and “bacteria” began to appear. From 2012 to 2016, the keywords “inflammation,” “insulin resistance” and “metabolism” began to appear. “metabolism” gradually became the focus of research, with *A. muciniphila* extensively studied for its potential in preventing and treating intestinal inflammation, diabetes, and related metabolic disorders. Between 2017 and 2020, “probiotics,” “metabolic syndrome” and “intestinal microbiota” have emerged as key areas of research, reflecting the further focus of research on the mechanisms by which *A. muciniphila* regulates metabolism and improves the gut microbiota. In 2018, the emergence of “supplementation” and “prebiotics” signaled the potential of *A. muciniphila* ‘s probiotic properties for nutritional interventions and clinical applications, and the role of *A. muciniphila* as a dietary supplement. *A. muciniphila* ‘s role as a dietary supplement is gradually being explored.

#### 3.5.4 Analysis of keyword emergence

**Figure 10:**
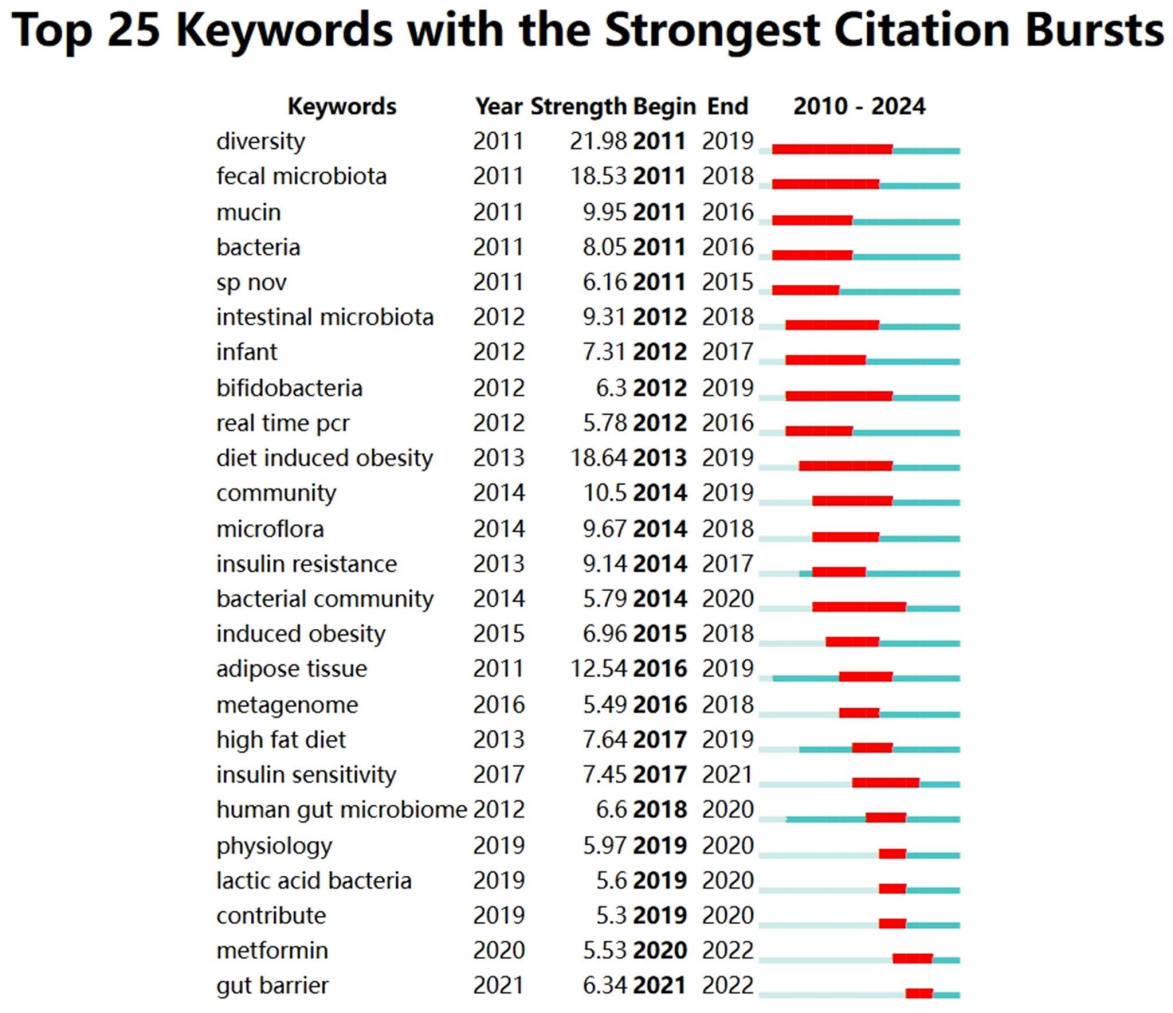
Keywords of *A. muciniphila* mutation map.

Figure 10 lists the 25 keywords with the strongest citation bursts between 2010 and 2024. The list of keywords in the figure shows the keywords with the strongest citation bursts between 2010 and 2024, “Year” indicates the year in which the keyword first appeared, “Strength” is the strength of the citation bursts, the larger the value indicates the stronger the citation bursts, “Begin” is the year in which the citation bursts started, and “End” is the year in which the citation bursts ended. The red part of the timeline indicates the active period of the citation outbreak, and the blue part indicates the existence period of the keyword in the whole time period.

The emergence of “diversity” (burst intensity 21.98) and “fecal microbiota” (burst intensity 18.53) in 2011 marks the beginning of the initial exploration of the gut microbiota through high-throughput sequencing or fecal transplantation. Between 2012 and 2016, research increasingly concentrated on “bifidobacteria” (burst intensity 6.3) and “diet induced obesity” (burst intensity 18.64), starting to focus on the role of various gut microorganisms and flora in the gut flora on metabolic health. Later on, the studies on “insulin resistance” (burst intensity 9.14), “insulin sensitivity” (burst intensity 7.45), and “high fat diet” (burst intensity 7.64) began to focus on the metabolic health of different species of microorganisms and flora in the gut flora. *A. muciniphila*, the sole representative of Bacillariophyta (phylum of warts and microfungi), was identified to enhance insulin sensitivity and alleviate insulin resistance, thereby advancing its study in metabolic research. In 2021, the outbreak of the keyword “gut barrier” suggests that researchers are focusing on the importance of *A. muciniphila* in maintaining gut barrier function and its potential role in preventing intestinal inflammation and related diseases.

#### 3.5.5 Analysis of citation emergence

**Figure 11:**
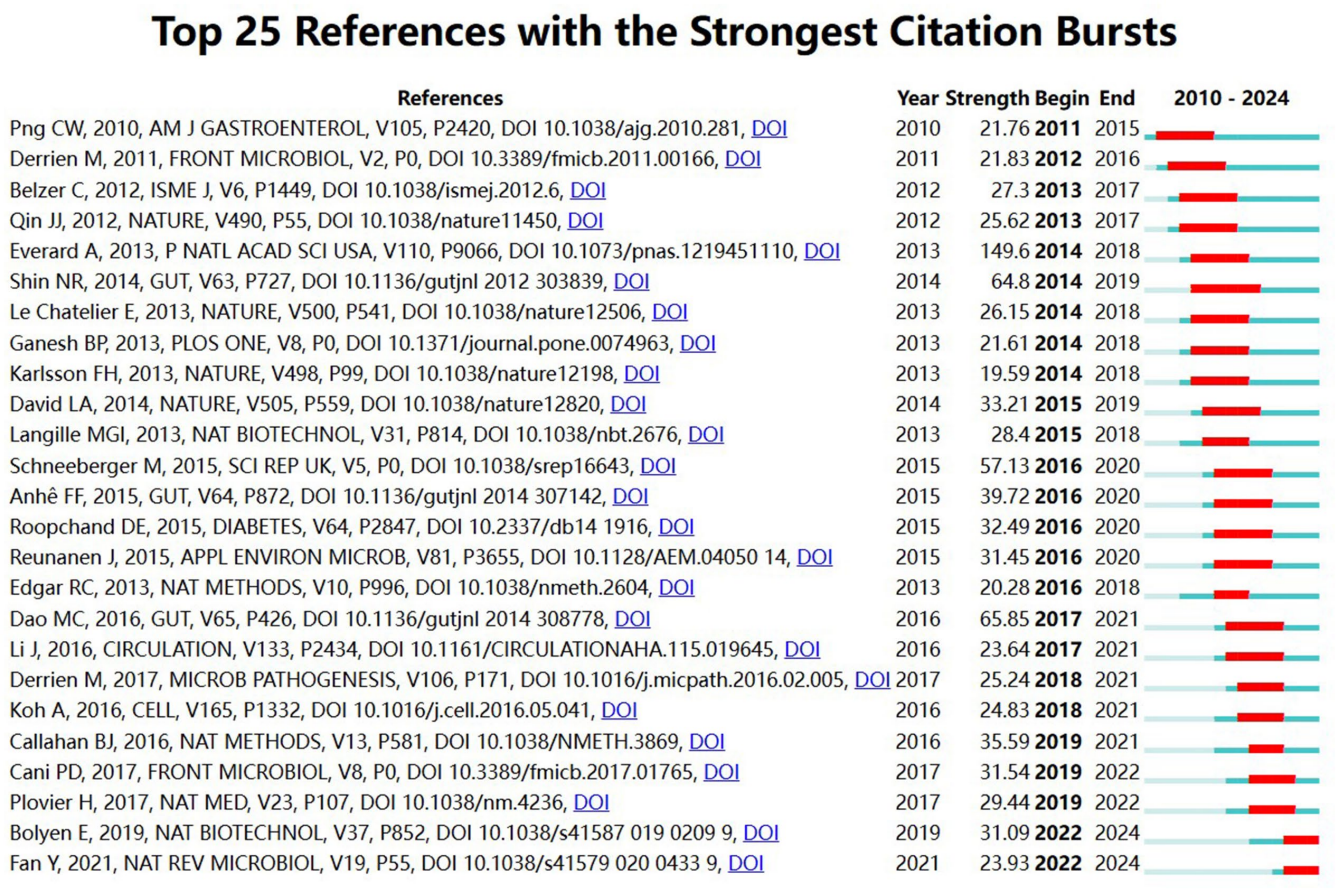
Map of reference hotspot year emergence for *A. muciniphila.*

The top 25 references with the highest number of article citations from 2010 to 2024 are shown in Figure 11, and citation frequency is an important indicator for assessing the importance of academic literature. The blue bars indicate when the references were published and the red bars indicate the emergence of citation frequency. The top 25 references include 16 experimental studies (9 animal studies, 6 clinical studies, and 1 *in vitro* study), 5 review articles, 2 methodological articles, and 2 newsletters, many of which are from top journals such as *Cell*, *Nature*, *Circulation*, and *Gut*, and most of which have citation bursts that lasted for a period of 5–6 years, with far-reaching academic impacts in the field of *A. muciniphila*. Among them, Everard et al. (2013) published in the journal *PANS* (IF = 9.1) with an outbreak intensity of 149.6, which found that *A. muciniphila* reduces the prevalence of obesity and type 2 diabetes, through an in-depth study of the mechanisms of interaction between *A. muciniphila* and intestinal epithelial cells. Endogenous cannabinoid levels in inflammation, intestinal barrier and intestinal peptide secretion could be controlled by *A. muciniphila* supplementation, thus reversing the metabolic disorders induced by high-fat diet (Everard et al., 2013). The literature by Dao et al. (2016) and Shin et al. (2014) were both published in *Gut* journal (IF = 23.0), with burst intensities of 65.85 and 64.8, respectively. Dao et al. through clinical study of dietary intervention in obese individuals, by analyzing *A. muciniphila* abundance, fecal microbial gene abundance, and bioclinical parameters before and after the intervention, it was found that the abundance of *A. muciniphila* was strongly correlated with healthy metabolic status such as weight loss and increased insulin sensitivity (Dao et al., 2016). Shin et al. showed that treatment with metformin significantly improved the high-fiber diet-fed mice’s glycemic status and showed higher abundance of the mucin-degrading bacterium *A. muciniphila* than controls, emphasizing that drugs favoring *A. muciniphila* species may be a potential therapeutic approach for type 2 diabetes (Shin et al., 2014).

## 4 Discussion

The present study systematically maps the research evolution of *A. muciniphila* through bibliometric analysis of the Web of Science database for the period 2010–2024. The analysis revealed a general trend of steady growth in research output related to *A. muciniphila* over the past 15 years. Since 2018, the number of publications has continued to show a rapid growth trend. The top contributing countries and institutions are China, the United States, South Korea, Spain, and Italy, with research centers such as the Chinese Academy of Sciences, Zhejiang University, the University of Copenhagen, and the University of Helsinki being the main contributors. Wei Chen, Willem de Vos, and Patrice D. Cani are the most influential scholars. The analysis of keywords and citations makes it possible to identify the most relevant research themes and their temporal evolution. The field exhibits a three-stage progression from basic knowledge to mechanism exploration to clinical translation: the early period (2010–2011) centered on basic concepts such as “intestinal flora” and “*A. muciniphila*,” focusing on identifying their taxonomic characteristics, which was accompanied by the development of high-throughput sequencing technologies (e.g., the emergence of the “diversity” and “fecal microbiota”), which pushed forward the initial knowledge of intestinal microecology; the middle period (2012–2016) *A. muciniphila* was linked to metabolic disorders, and the key role of *A. muciniphila* in improving insulin sensitivity and alleviating obesity-associated metabolic disorders was clarified through the emergence of keywords such as “insulin resistance” “high-fat diet”; in the near future (2017–2024), it enters the phase of mechanism deepening and transitioning to clinical application, with the continued activation of keywords such as “probiotic”, “supplementation”, “intestinal”, and other keywords continue to be active (Wu et al., 2022), reflecting the potential value of this bacterium in the regulation of metabolic pathways, maintenance of intestinal mucosal function and nutritional intervention. Notably, the core contributions of the 16 highly mutated papers identified in this study focused on revealing the metabolic protection of *A. muciniphila* through the regulation of endogenous cannabinoid system, enhancement of the intestinal barrier function, and remodeling of the microbial-brain axis, which provided important theoretical support for the development of subsequent precision intervention strategies. Current research focuses on the molecular biology of *A. muciniphila*, such as its role in intestinal barrier maintenance, immune response, and its potential for regulating and treating digestive and metabolic diseases, such as cancer, fatty liver disease, inflammatory bowel disease, etc., through bile acid metabolism, extracellular vesicles, and insulin resistance.

Bile acids, as an important component of bile, synthesized by the liver and stored in the gallbladder, enter the intestines with the diet. There, they interact with specific intestinal flora to become secondary bile acids, which are absorbed in the terminal ileum through apical sodium-dependent bile acid transporters. Most bile acids return to the liver via the portal vein, completing the hepatic-intestinal cycle, while a small portion crosses the blood–brain barrier through somatic circulation to enter the brain (Bhargava et al., 2020). The intestinal flora modulates bile acid composition and structure, affecting their physicochemical properties and biotoxicity. It converts primary bile acids to secondary ones and activates bile acid receptors like farnesoid X receptor (FXR) and takeda G protein-coupled receptor 5‌ (TGR5) (Chand et al., 2017; Bao et al., 2021; McMillin et al., 2015; Guo et al., 2016). In 2020, the journal Nature published several reports on bile acid metabolism, and it became another “hot” metabolite in the intestinal flora after short-chain fatty acids (Ridlon and Gaskins, 2024). According to Nature Medicine, *A. muciniphila* exerts a biological effect in mice by either colony colonization or oral administration, and the mechanism is likely to be the restoration of secondary bile acids in the intestinal tract (Bárcena et al., 2019). Further studies have shown that gavage of *A. muciniphila* in obese mice improves abnormal bile acid metabolism and helps to remodel the composition of the intestinal microbiota (Rao et al., 2021). Currently, most studies focus on the mechanism of *A. muciniphila* regulating bile acid metabolism in fatty liver disease. For example, *A. muciniphila* remodeled the structure of bile acids by regulating the intestinal FXR-recombinant fibroblast growth factor 15 (FGF15) axis, reduced secondary bile acids (e.g., deoxycholic acid and lithocholic acid) in the cecum and liver, and ameliorated metabolism-associated steatohepatopathies in high-fat diet-induced obesity mouse models (Wu et al., 2023). A recent study in *Cell* demonstrated that the novel bile acid 3-succinylcholate can reverse metabolic dysfunction-associated steatohepatitis in mice. This effect is achieved by promoting *A. muciniphila* growth, improving intestinal barrier integrity, and reducing chronic low-level inflammation, revealing a mechanism by which intestinal commensals alleviate metabolic dysfunction-associated steatohepatitis through secondary bile acid biosynthetic pathways (Nie et al., 2024).

Extracellular vesicles, ranging from 20 to 400 nm in diameter, are lipid bilayer nanostructures secreted by both Gram-negative and Gram-positive bacteria within the intestinal microbiota. These vesicles facilitate host-bacterial communication by transferring genetic material and proteins to the host or by directly interacting with immune and epithelial cells (Ellis and Kuehn, 2010; Lee et al., 2009; Kuehn and Kesty, 2005). *A. muciniphila*-derived extracellular vesicles are rich in bioactive macromolecules that enhance tight junctions and mucus expression, thereby playing a crucial role in regulating intestinal barrier function (Ashrafian et al., 2019; Chelakkot et al., 2018). Wang et al. found that extracellular vesicles from *A. muciniphila* can penetrate intestinal epithelial cells, enhancing tight junctions, boosting mucus expression, reducing intestinal permeability, and thus preserving intestinal wall integrity, which may help prevent inflammatory bowel disease (Wang et al., 2023). Furthermore, *A. muciniphila* and their extracellular vesicles alleviated non-alcoholic fatty liver disease (NAFLD) by inhibiting inflammation in a high-fat diet/carbon tetrachloride-induced liver injury model in mice (Raftar et al., 2022). The biocompatibility of extracellular vesicles, as natural vesicles at the nanoscale, makes them drug delivery vehicles that can specifically target cells and tissues (Wang et al., 2022; Zou et al., 2021), providing unique advantages for cancer therapy. *A. muciniphila*’s research in cancer has progressively increased from 2021 to the present day and has been mostly combined with extracellular vesicles, working on lung, colorectal, and prostate cancer therapeutic exploration (Derosa et al., 2022; Luo et al., 2021; Jiang et al., 2023). *A. muciniphila* can enhance immune checkpoint inhibitors’ effectiveness by interacting with immune cells in the tumor microenvironment and lymphoid tissues via secreted extramembranous vesicles. For example, *A. muciniphila*-extracellular vesicles improved the effectiveness of anti-PD1 therapy in colorectal cancer by reversing the tumor’s resistance to PD-1, achieved through the up-regulation of beneficial gut microbiota that enhance PD-1-based immune response (Wang et al., 2023). In addition, a study in Nature Medicine sequenced the macrogenomics of 338 advanced non-small cell lung cancer patients treated with immune checkpoint inhibitors, prospectively validating in a multicenter setting that fecal *A. muciniphila* was linked to higher remission rates and overall survival. This association was further confirmed using a mouse model to explore *A. muciniphila* and its ecosystems’ impact on clinical outcomes. The study identified a link between clinical outcomes in patients and resistance to anti-PD-1 antibodies in mice, which was associated with a lack of *A. muciniphila* in their fecal microbiota (Derosa et al., 2022).

Although *A. muciniphila* has not been highlighted in the field of neurological diseases in the Hot Spot Analysis section due to the limitation of time and the number of literatures, the author found that recent studies indicate that *A. muciniphila* plays a role in ameliorating various neurological diseases. Oral administration of *A. muciniphila* improves hippocampus-dependent learning and memory in mice on an early high-fat diet, decreases proinflammatory cytokine expression, and enhances neuronal development and synaptic plasticity (Yang et al., 2019). A study in *Nature* exploring the potential role and regulatory mechanisms of gut microbiota and metabolites in the disease course of amyotrophic lateral sclerosis mice showed that mice administered *A. muciniphila* showed potential therapeutic effects in slowing down the symptoms and improving the progression by accumulating the associated nicotinamide in the central nervous system (CNS) (Blacher et al., 2019). Vallio et al. compared anti-*A. muciniphila* IgG levels in the cerebrospinal fluid between multiple sclerosis patients and healthy individuals, found that anti-*A. muciniphila* IgG levels were notably elevated in multiple sclerosis (Vallino et al., 2020).

We suggest the following directions to deepen the exploration of *A. muciniphila* research priorities and urgent bottlenecks: (1) Research on the molecular mechanism of *A. muciniphila*: The key active component of *A. muciniphila*, the outer membrane protein Amuc_1100, has been shown to have anti-inflammatory, metabolism-regulating and immune-regulating functions, but its specific pathways of action (e.g., the TLR2/NLRP3 pathway) still need further validation. Secondly, how *A. muciniphila* interacts with host immune cells (e.g., macrophages, Treg cells) through metabolites (e.g., short-chain fatty acids, bile acids, endogenous cannabinoids) needs to be explored in-depth by combining single-cell sequencing and metabolomics. Meanwhile, the specific active components in *A. muciniphila* exosomes and the molecular mechanisms of intestinal barrier and immunomodulation are not completely clear. (2) Safety assessment of clinical applications: Although currently *A. muciniphila* has shown safety in animal models, there are limited clinical safety data, and its potential risks in different populations (such as active inflammatory bowel disease) have not been fully evaluated. The safe dosage, especially the side effects of long-term use, urgently need to be clarified. There is a need to conduct large-scale clinical trials to verify its long-term safety and efficacy in metabolic diseases, inflammatory bowel disease and other diseases, and to establish an optimized regimen based on disease type and patient stratification. (3) Standardized production: as *A. muciniphila* requires harsh culture conditions, large-scale fermentation technology is still immature, and there are technical bottlenecks in maintaining the stability and activity of inactivated strains, including the maintenance of anaerobic conditions, the stability of lyophilized preparations, and other industrial culture technology is still facing challenges. It is crucial to promote the conversion of *A. muciniphila*-related patents (e.g., strain preservation, double emulsion encapsulation, metabolite development) into actual products (Nugroho et al., 2017). (4) Cross-disease applications and optimization of dosing regimens: First, in metabolic diseases such as obesity, diabetes, and non-alcoholic fatty liver disease, it is necessary to further clarify the specific path of regulating intestinal flora diversity and host metabolism. Among them, it is noteworthy that clinical trials have confirmed that a 3-month intervention with a pasteurized form of *A. muciniphila* supplementation (10^10^ CFU/day) resulted in a significant increase in insulin sensitivity index of about 30%, as well as improvements in body weight, cholesterol and triglyceride levels (Depommier et al., 2019). It is recommended to focus on the development of precise strain formulations targeting insulin resistance phenotypes and to explore synergistic effects with GLP-1 receptor agonists (Chen et al., 2022; Zhao et al., 2024). Second, animal experiments have shown that the *A. muciniphila* outer membrane protein Amuc_1100 can be used as an intestinal barrier repair agent to repair intestinal epithelial tight junctions (Plovier et al., 2017), which can advance the engineering modification of this protein for the development of gastric acid-resistant oral nanodelivery systems (e.g., in combination with chitosan microcapsule technology) (Zou et al., 2022). In addition, *Science* has reported that the abundance of *A. muciniphila* in the intestines of cancer patients (including lung, kidney, and bladder cancers, etc.) who were effective in anti-PD-1 therapy was significantly higher than that of patients who were ineffective; furthermore, after transplanting the intestinal flora containing a high abundance of the *A. muciniphila* population into sterile mice, the therapeutic response of the mice to the PD-1 inhibitor was significantly enhanced, the increased infiltration of CD8 + T cells in the tumor microenvironment and elevated anti-tumor effects (Routy et al., 2018). Therefore, it remains to be explored whether *A. muciniphila* is expected to be used as an adjuvant for tumor immunotherapy, as well as the synergistic effects of *A. muciniphila* in tumor immunotherapy, such as in combination with immune checkpoint inhibitors and chemotherapeutic agents.

This pioneering bibliometric analysis of *A. muciniphila* highlights the significance of the gut flora *A. muciniphila*, focusing on its current status and research hotspots, despite certain limitations. The study was limited to visualizing and analyzing literature from the Web of Science database due to software constraints. Although Web of Science is a reputable source, literature exclusive to other databases like PubMed and Embase may have been omitted (Guo et al., 2022). Secondly, only English language literature was included in our study, and there is a possibility of omission of non-English language literature on related topics.

## 5 Conclusion

In this study, we systematically analyzed the research trajectory of *A. muciniphila* from 2010 to 2024 by bibliometric methods, revealing that the research on *A. muciniphila* has moved from colony description and functional validation to the analysis of the deeper mechanisms of colony-host interactions and the multidimensional evolution of precision medicine applications. Currently, the translational potential of *A. muciniphila* coexists with challenges, and it is still necessary to solve the existing bottlenecks through technological innovation, data fusion, and interdisciplinary collaboration to further elucidate its precise roles in human health and to lay the foundation for the development of novel microbe-based therapies. Therefore, it is recommended to focus on the following directions in the future:

1. Interdisciplinary mechanism deepening: combining multi-omics technology to analyze the interaction between *A. muciniphila* and host signaling pathways, and clarifying its deeper mechanism in chronic diseases.
2. Precise intervention strategies: develop new probiotics or engineered bacteria that can target and regulate the abundance of *A. muciniphila*, break through the difficulties in formulation stability caused by its strictly anaerobic characteristics, and explore personalized supplementation solutions.
3. Clinical translation: Expanding population-based cohort studies to validate the potential of *A. muciniphila* as a disease biomarker, and conducting long-term safety assessment to promote its clinical application in metabolic syndrome, inflammatory bowel disease and other areas.

The field is becoming more and more interesting and recognized. We strongly advocate for enhanced international collaboration among countries, institutions, and researchers to conduct comprehensive basic and clinical research on *A. muciniphila* across a wider range of diseases.

## Data Availability

The original contributions presented in the study are included in the article/supplementary material, further inquiries can be directed to the corresponding authors.
